# An Overview on the Use of miRNAs as Possible Forensic Biomarkers for the Diagnosis of Traumatic Brain Injury

**DOI:** 10.3390/ijms24076503

**Published:** 2023-03-30

**Authors:** Giuseppe Davide Albano, Chiara Stassi, Antonina Argo, Stefania Zerbo

**Affiliations:** Section of Legal Medicine, Department of Health Promotion, Mother and Child Care, Internal Medicine and Medical Specialties, University of Palermo, 90129 Palermo, Italy

**Keywords:** traumatic brain injury, miRNA, forensic, autopsy, biomarkers

## Abstract

Determining the cause of death is one of the main goals of forensic pathology. However, conditions can occur in which common approaches—external inspection, autopsy, histology, etc.—might not be conclusive. With the advancement of molecular biology, several investigative techniques have been developed over the years, and the application as approaches complementary to routine procedures has proved useful in these cases. In this context, microRNA (miRNA) profiling has attracted increasing interest due to these molecules’ ability to regulate physiological and pathological processes. The evidence of differential miRNA expression in both animal models and human samples of traumatic brain injury (TBI) has laid the basis for comprehension of the underlying pathophysiological mechanisms, thus allowing us to identify some of them as possible TBI diagnostic biomarkers. The present narrative review aims to explore the primary miRNAs involved in the mechanisms underlying TBI, which could be considered for future evaluation as possible markers in a post mortem setting.

## 1. Introduction

Due to the comprehensive information they can provide, molecular investigations are raising an increasing interest for their possible application to the forensic field. In this regard, several branches, such as forensic genomics, transcriptomics, and proteomics, have frequently proved useful as complementary approaches to routine procedures by helping define the genetic and biochemical bases underlying the cause of death [1].

The ability to regulate physiological and pathological processes, together with the evidence of tissue-specific expressions, has opened up a wide range of possible applications for microRNA profiling in forensic diagnostics over the last decade. In RNA profiling, microRNAs (miRNAs) have been investigated lately as possible forensic biomarkers, mainly due to their small size, making them available to be recovered even in highly degraded samples [2,3]. miRNAs are single-stranded, 18- to 24-nucleotide-long fragments whose contribution to the regulation of several biological processes is explicated at the post-transcriptional level by complementary binding sequences of mRNA so that gene expression can be silenced through either mRNA degradation or impaired protein synthesis [4]. Indeed, since the first suggestion of their application for body fluid identification [5], the potential of miRNA profiling has been applied to other forensic issues, including post-mortem interval (PMI) estimation, the vitality of wounds, brain injury related to aging, drug abuse, and stroke, and drowning and sepsis diagnosis of death [3,6,7].

To investigate the role of miRNAs in the pathophysiological events underlying traumatic brain injury (TBI), due to the similarities between human and animal function mechanisms, several researchers have engaged in the evaluation of the changes in their expression profile in experimental rodent models, by inducing TBI with either weight drop, fluid percussion, or controlled cortical impact [8]. The results obtained on these pre-clinical models led to identifying groups of miRNAs that could be identified as TBI biomarkers per se, but also showed time- and severity-dependent expression changes (miR-132, miR-21 and miR-30a, miR-let-7i are among the most evaluated) [9,10,11]. One of the most studied miRNAs in pre-clinical models is miR-21, whose up-regulation following TBI has been found to lead to the inhibition of apoptosis through targeting Bcl2, PTEN, and CDP4, as well as to the promotion of the outgrowth of neuronal axons by activating Ang-1/Tie-2 and Akt signaling, thus representing a potential mechanism by which the brain attempts to limit trauma-related neuronal destruction [12,13,14,15].

The importance of such findings lies in translating them into human models. A few works have been recently produced in which miRNA expression profiles have been evaluated within a clinical context, this leading not only to the validation of specific miRNAs as TBI diagnostic markers but also to the correlation of their differential expression to severity and prognosis; in addition, the comprehension of the mechanisms by which they act within specific cellular pathways has also laid the basis for their possible use for therapeutic purposes. Significant evidence concerning the role of miRNAs as TBI markers in a forensic context is still lacking; in this light, the present narrative review aimed to explore the primary miRNAs involved in the mechanisms underlying TBI that could be considered for future evaluation as possible markers in a post mortem setting. With this purpose in mind, although enlightening, experimental investigations on animal models have been excluded; since the field of application we want to explore is the possible use of miRNAs in a forensic setting, we preferred to focus on works carried out on human samples in a clinical context, which might be proposed as an equivalent comparative system for future post mortem investigations.

This research, performed using the PubMed and Scopus databases, was carried out according to the following inclusion criteria: English language; evaluation of miRNA expression on human samples; miRNA detection carried out on serum and plasma samples; and miRNAs that show an increase in cases of TBI. The articles selected included reviews, original articles, and prospective studies; references from the chosen articles were also evaluated for possible inclusion. Exclusion criteria were languages other than English and unavailability.

## 2. Traumatic Brain Injury: Definition and Pathophysiological Role of miRNAs

Traumatic brain injury (TBI) is an impairment of cerebral functions due to direct or indirect mechanical insults, such as blasts, assaults, collisions, penetrative injuries, etc. It can be classified as a primary or secondary injury; the first one consists of the acute pathological changes induced by an external force at the (time) of the impact, which are mainly represented by internal hemorrhages, brain contusion, and axonal and vascular damage; the second one comprises all those pathological processes leading to further impairment of the neurological functions (oxidative stress, glutamate excitotoxicity, Ca^2+^ overload, inflammatory response). With an annual incidence of around 50 million individuals, TBI represents one of the leading causes of disability worldwide since the related neurodegeneration increases the risk of developing chronic behavioral, cognitive, and physical impairment, as well as dementia, Alzheimer’s disease, and Parkinson’s disease [8,16,17,18,19].

TBI severity can be classified as mild, moderate, or severe according to the Glasgow Coma Scale, a scoring system that consists of the evaluation of eye, motor, and verbal responses; a score of 13–15 corresponds to a mild TBI (mTBI); a 9–12 score corresponds to a moderate TBI; and a ≤8 score identifies a severe TBI. Other parameters contributing to better defining the severity and prognosis of TBI include clinical outcome, loss/alteration of consciousness, impaired mental state, and brain alterations detected by imaging [8,19].

From a diagnostic point of view, imaging represents the gold standard. Computed tomography (CT) allows the identification of the injured cerebral areas and the extent of injury, thus guiding the most appropriate clinical or surgical management. In selected cases, MRI can also provide important information due to its better tissue contrast and increased sensitivity compared to CT. Nonetheless, mTBI is frequently associated with a lack of visible signs of head impact (bleeding, lacerations) on neuroimaging, thus making it difficult to either achieve a correct diagnosis or make a prognostic evaluation [8,16,17]. Therefore, to overcome such limits, due to the knowledge of the pathophysiological mechanisms underlying TBI (release of cytokines, chemical mediators, and neurotransmitters; NO-dependent and Ca^2+^-dependent induction of apoptosis), several researchers have engaged over time in the identification of fluid biomarkers of injured axons, neuronal and glial cells, as well as inflammation biomarkers [17,20].

Since almost 70% of miRNAs are reported to be expressed in the central nervous system—where they guide all stages of neurodevelopment and function—those involved in the molecular pathways activated by TBI have been gaining attention over the last decade as possible fluid biomarkers [17].

The complex TBI-related molecular network in which soluble miRNAs are involved was carefully elucidated in a meta-analysis by Cente et al. [19], who made use of bioinformatic systems to identify the target genes for deregulated miRNAs isolated from plasma, serum, and cerebrospinal fluid, and the related signaling pathways, to link severe TBI to the pathogenesis of neurodegeneration. As a result, they found that the miRNA-targeted genes following severe TBI were involved in a great variety of processes, such as brain development, neurogenesis, myelinization, oligodendrocyte differentiation, regulation of synaptic plasticity, axon guidance, and regulation of inflammatory genes. As for the molecular pathways activated, one of the most significant was identified in the activation of BDNF/TrkB signaling downstream of the PI3K/AKT/MAPK pathway, with a neuroprotective role. Another dominant pathway, shared among all examined biofluids, was the one involving WNT/β-catenin and Notch signaling, with a neurodegenerative and reparative role. As expected, inflammatory pathways also figured among the dominant ones, with a significant involvement of IL-2, TGF-β, TLR, and integrin signaling, which were reported to play neuroprotective roles, regulate the pro-inflammatory activity of microglia and astrocytes, and suppress neuro-inflammation and blood–brain barrier disruption following TBI. Center et al. also found that most of the identified pathways were shared among the tested biofluids. Such evidence suggested the activation of a complex interaction between the brain, periphery, and immune system, thus confirming the role of miRNAs in the neuropathophysiology of TBI and their possible value as diagnostic and prognostic biomarkers [19].

## 3. Traumatic Brain Injury and Exosome-Derived miRNAs

Extracellular vesicles (EVs) are subcellular particles playing an important role in intercellular communication. Their structure consists of a lipid bilayer membrane whose cargo—originated from the parental cell—is variably represented by lipids, proteins, DNA, mRNAs, miRNAs, non-coding RNAs, and organelles. Based on origin and dimensions, EVs can be classified into three main subtypes: exosomes (Exos, 10–100 nm diameter) that originate from the endosomal/multivesicular body (MVB) system and are stored inside the cell before their release; microvesicles (MVs, 100–1000 nm) that originate from a budding process of the plasma membrane; and apoptotic bodies (1–5 nm) that are generated from apoptotic cells and contain degradation products.

All brain cells produce EVs, including neurons, astrocytes, microglia, and oligodendrocytes, but their content is highly variable depending on the external signals. Within a neurological context, they play a key role in modulating synaptic activity and neuronal communication, thus contributing to the pathogenesis of several neurodegenerative processes, including those underlying TBI.

Among EVs, exosomes are of particular interest due to the ability of their miRNA cargo to modulate the gene expression pattern in recipient cells and induce systemic inflammation [21,22,23]. Such a role is highlighted in the work of Long et al. [24], who carried out an experimental study in which TBI brain extracts were used to stimulate the production of exosomes in primary cultured astrocytes; once detected by immunofluorescence, exosomes were separated from astrocytes and added to primary cultured microglia. Subsequent immunofluorescence, qRT-PCR, and western blotting allowed not only confirmation of the exosome uptake by microglia but also the induction of a gene expression pattern consistent with a polarization of microglia into an M2 phenotype with an anti-inflammatory role. A subsequent miRNA microarray analysis of exosomes derived from astrocytes showed a significant up-regulation of 135 different miRNAs, out of which the most represented appeared to be miR-873a-5p, involved in the NF-κB signaling pathway leading to microglial activation. Based on these results, miR-873a-5p expression was evaluated by qRT-PCR in clinical specimens of damaged brain tissue obtained from 15 patients who underwent neurosurgery. Brain tissue samples were all collected three days after the TBI occurrence and consisted of either necrotic brain tissue or severe edema around the necrotic lesion. As a result, miR-873a-5p expression appeared significantly higher within necrotic areas than in the edematous areas. Subsequent in vitro experiments showed that miR-873a-5p suppresses pro-inflammatory factors and promotes the release of anti-inflammatory factors from the microglia by inhibiting both ERK phosphorylation and the NF-κB signaling pathway. Taken together, these findings suggested the role of miR-873a-5p as a possible TBI marker and, simultaneously, as a therapeutic target for improving cerebral injuries and impaired neurological functions.

Vorn et al. [25] assessed the expression levels of plasma exomiRNAs in 29 subjects with a history of chronic mTBI compared to 11 healthy controls. As a result, 25 different plasma exomiRNAs appeared differentially expressed in chronic mTBI compared to healthy controls; among them, only 4 were up-regulated (hsa-miR-520e, hsa-miR-499b-3p, hsa-miR-520b, hsa-miR-4488). Further bioinformatic investigations helped explicate the molecular mechanisms underlying the impairment of brain function after mTBI; indeed, 14 exomiRNAs were related to neurological disease, 23 were related to organismal injury and abnormalities, and 13 were related to psychological disease.

## 4. Traumatic Brain Injury and Circulating miRNAs

Compared to the well-known protein TBI biomarkers, circulating miRNAs might be preferable due to their specific characteristics. First of all, their small sizes allow higher stability even in highly degraded samples. Secondly, the high tissue-specific expression gives them a higher sensitivity to the pathology examined. In addition, due to their action at a post-transcriptional level, they can be detected in the early stages of a disease, long before the effects of downstream protein expression are observed. For these reasons, several works have been produced to evaluate the different miRNA profiles in fluid samples of TBI models compared to controls [26]. We present here a series of studies that aimed to find possible TBI miRNA markers in human fluid samples.

Redell et al. [27] examined miRNA expression in plasma samples obtained from 15 severe TBI patients compared to healthy volunteers. Preliminary microarray analysis revealed an up-regulation of 33 miRNAs and a down-regulation of 19 other miRNAs; five of these—miR-16, miR-20a, miR-92a, miR-638, and miR-765—were then selected using known expression patterns potentially involved in TBI pathophysiology. Subsequent qRT-PCR carried out on samples collected within 24 h from TBI allowed confirmation of the potential value of miR-16, miR-92a, and miR-765 as diagnostic biomarkers. The comparison between TBI patients and non-TBI orthopedic injury controls revealed that miR-16 and miR-92a expression was significantly lower in TBI patients than in orthopedic controls but increased dramatically in mild TBI patients compared to healthy volunteers; in addition, the same two miRNAs could not be used to differentiate between mild TBI patients and orthopedic controls.

Bhomia et al. [28] compared serum miRNA profiles between severe TBI patients (sTBI), mild to moderate TBI (mmTBI) patients, orthopedic injury controls, and healthy volunteers using qRT-PCR analysis. They observed an up-regulation of 39 miRNAs in mmTBI, 37 miRNAs in sTBI, and 33 miRNAs in an orthopedic injury group compared to control samples. Ten of these (miR-151-5p, miR-22 195, miR-20a, miR-30d, miR-328, miR-362-3p, miR-451, miR-486, miR-505*, and miR-92a), which appeared up-regulated in both mild/moderate and severe TBI patients, were selected for further validation in CSF, which showed that only 4 of them (miR-328, miR-362-3p, miR-451, and miR-486) were also up-regulated in this sample. Bhomia et al. further showed that eight of the ten miRNAs (miR-195, miR-30d, miR-451; miR-92a, miR-486, miR-505, miR-362, and miR-20a) were significantly increased in TBI patients with abnormal CT scans (*n* = 12) compared to TBI patients with regular CT scans (*n* = 19).

In their work, Di Pietro et al. [29] evaluated the circulating miRNAs in serum samples collected one day and 15 days after injury from five mild TBI and five severe TBI patients. They compared these to samples obtained from five healthy volunteers. Array analyses revealed significant changes in the differential expression of 10 miRNAs at one day and 15 miRNAs at 15 days for mTBI samples compared to healthy controls; among these, only five (miR-425-5p, miR-126*, miR-144*, miR-590-3p, and miR-624) were similar between the two time points in mTBI patients. Analog significant differential expression was observed for 19 miRNAs at one day and 22 miRNAs at 15 days in sTBI samples compared to healthy controls, particularly miR-21, miR-335, miR-190, miR-193a-5p, miR-144*, and miR-625*. To identify miRNA changes that could differentiate between mild and severe TBI, Di Pietro et al. selected those miRNAs appearing specifically altered in mTBI (miR-425-5p and miR-502) or sTBI (miR-21 and miR-335) patients and further validated them in a total of 120 patients divided into four groups: 30 mTBI, 30 sTBI, 30 extra-cranial injury (EC) controls, and 30 healthy patients; all serum samples were collected at T0, T4–12 h, T48–72 h, and 15 days. Among the examined miRNAs, only miR-21 was significantly up-regulated in sTBI patients compared to mTBI, EC controls, and healthy patients, and also correlated with age, CT lesions, and the Injury Severity Score.

Qin et al. [30] performed an initial microarray assay on plasma samples obtained from a total of 90 TBI patients, which, compared to a group of 30 healthy controls, showed an up-regulation of 65, 33, and 16 miRNAs, as well as a down-regulation of 29, 27, and 6 miRNAs, in patients with mild, moderate, and severe TBI, respectively. Among these, 13 miRNAs (7 up-regulated and 6 down-regulated) were common to all TBI groups. Subsequent pathway enrichment analyses showed that they shared commonly activated pathways, including the p53, mTOR, and TGF-β signaling pathway, SNARE interactions in vesicular transport, and the neurotrophin signaling pathway. Qin et al. then selected seven candidate miRNAs (miR-6867-5p, miR-3665, miR-328-5p, miR-762, miR-3195, miR-4669, and miR-2861) to be validated using qRT-PCR on a total of 100 separate and independent samples (25 in each group). All miRNAs appeared significantly up-regulated in all three TBI groups when compared to control samples, but the levels of miR-3195 and miR-328-5p were higher in the severe TBI group than in the mild and moderate TBI groups; in addition, miR-6867-5p levels in moderate and severe TBI groups were higher than those in mild TBI.

Time-dependent miRNA expression in cases of severe TBI was evaluated by Ma et al. [31], who analyzed the serum obtained from a total of 20 patients. An RT-PCR analysis was performed, and changes in miRNA profiles were observed at 2, 12, 24, 48, and 72 h, with the following results: miR-18a, miR-203, miR-146a, miR-149, miR-23b, and miR-let-7b showed a >10-fold increase at 12 h compared to the 2 h time-point; all the previously cited except miR-18a showed the same magnitude of increase also at 24 h; miR-181d, miR-29a, and miR-18b showed a >5-fold increase at 48 h; miR-203, miR-146a, and miR-149 showed a >5-fold increase after 72 h. The use of bioinformatic tools helped determine that all the differentially expressed miRNAs were involved in pathways mainly related to cell proliferation, apoptosis, differentiation, inflammatory response, and collagen formation.

Yan et al. [32] evaluated differentially expressed miRNAs between mild, moderate, and severe TBI. An initial array to evaluate the levels of 754 serum miRNAs was performed in two pooled samples of 15 sTBI patients and 15 healthy controls, identifying 19 up-regulated miRNAs in the sTBI group with unfavorable outcomes compared to the control group. Next, 12 of these 19 miRNAs were selected to be validated by qRT-PCR in the serum samples of a larger cohort consisting of 81 sTBI patients, 81 mTBI patients, and 82 healthy controls. As a result, seven miRNAs (miR-103a-3p, miR-219a-5p, miR-302d-3p, miR-422a, miR-518f-3p, miR-520d-3p, and miR-627) appeared significantly up-regulated in both sTBI and mTBI patients compared to controls, and among these, miR-219a-5p, miR-422a, and miR-520d-3p levels appeared significantly higher in sTBI patients compared to mTBI patients. Yan et al. also investigated the correlation between the expression levels of the seven identified miRNAs with CT lesions; with this aim, 26 TBI patients without head CT and 136 TBI patients with lesions on head CT were analyzed. As a result, miR-103a-3p, miR-219a-5p, miR-302d-3p, miR-422a, and miR-627 levels were significantly higher in TBI patients with lesions than in those without lesions on head CT. Further bioinformatic analyses highlighted the role of up-regulated miR-219a-5p in the inhibition of CCNA2 and CACUL1 expression, thus contributing to the regulation of Akt/Foxo3a and p53/Bcl-2 signaling pathways in neuronal apoptosis activation.

Lastly, Schindler et al. [33] engaged in the evaluation of the levels of six miRNAs (miR-9-5p, miR-124-3p, miR-142-3p, miR-219a-5p, miR-338-3p, and miR-423-3p) in blood samples obtained within six hours after trauma from 33 patients, divided into three groups: severely injured patients without TBI (PT), those with severe TBI (PT + TBI), and patients with isolated TBI (isTBI). The results showed that miR-9-5p, miR-142-3p, and miR-219a-5p could not be detected in any group, while miR-338-3p levels did not show any change between all trauma groups. Interesting results were obtained for miR-423-3p, whose expression significantly increased in patients with severe isTBI, followed by PT + TBI, compared to PT patients without TBI; statistical analyses further showed that miR-423-3p levels positively correlate with TBI severity and risk of mortality, leading to the conclusion that it could represent a promising biomarker to identify severe isolated TBI. The main findings of the reviewed articles are summarized in Table 1.

## 5. Advantages and Limits for Possible Forensic Applications

A correct post-mortem diagnosis of TBI needs a proper interpretation of the findings from different investigations, including external inspection, post-mortem radiology, autopsy, and histology as the routine gold standard procedures, and ante mortem data whenever available. Nonetheless, several conditions exist in which such approaches alone might not be conclusive, especially in cases in which other neuropathological conditions (ischemia, neurodegeneration, etc.) may have contributed to the death or in which some signs might be variably interpreted (e.g., in the absence of other traumatic signs, brain bleeding can either be interpreted as hypostatic or related to a traumatic subarachnoid or parenchymal hemorrhage); additional challenges are to be faced in cases of an advanced state of decomposition, where radiological, macroscopic, histological, and toxicological analyses cannot provide useful information [20,34,35,36].

In these scenarios, implementing new, innovative approaches in the forensic field becomes essential. Among these, the evaluation of TBI-related changes in miRNAs expression profiles has lately been attracting attention. Circulating miRNAs are preferable to other protein biomarkers for their intrinsic characteristics: higher stability even in highly degraded samples due to their small sizes; high stability even at extreme temperatures, pH conditions, and chemical treatments; transportation within lipoprotein complexes and RNA-binding proteins in extracellular vesicles, which preserves them from endogenous RNase activity [3,26,37]. In addition, unlike most proteins, brain-specific miRNAs can be easily evaluated in body fluids following injury since, due to their small sizes, they can cross the blood–brain barrier via microvesicles, exosomes, and lipoprotein carriers [37].

Depending on the desired information, miRNA profiling can be performed relying on different approaches, each easy to perform and providing high sensitivity and reproducibility. These include microarray and/or NGS when simultaneous detection of hundreds of low copy number miRNAs is requested, or qRT-PCR when the aim is to analyze only a few selected miRNAs [2,3,19].

Although such characteristics make miRNAs promising biomarkers in a post mortem setting and in a clinical one, some limits need to be considered. One of them is related to the lack of uniform and validated protocols on a global level, which prevents a comparison of the results between different laboratories. Another issue concerns the need for proper endogenous controls and normalization procedures that evaluate miRNA fold changes [2,3,19,26,37]. Last, but not least, the impact of demographic characteristics, such as age, sex, and body mass index, in miRNA profile variability should be considered [26].

Other limits are related to the samples used. Although plasma and serum, being easily accessible, represent the most suitable matrices for miRNA evaluation in a post mortem setting, the implementation of uniform procedures should be promoted to assess the sample quality before miRNA profiling: indeed, fibrinogen in plasma samples can be a source of contamination affecting the extraction quality, while serum clots can alter the actual miRNA profile [19,26,37]. Both advantages and limits are summarized in Table 2.

Although several TBI-related miRNAs have been identified that could be suitable for evaluation in a post mortem setting for diagnostic purposes, a great deal of work is yet to be performed to produce reliable protocols for obtaining uniform and comparable data between laboratories worldwide. Studies with larger samples are mandatory to confirm literature data regarding the role of miRNA in TBI diagnosis and to use these molecules as biomarkers in forensic investigation. The changing expression patterns of several miRNAs according to different neurological conditions highlight a possible role of miRNAs as a diagnostic tool in the medico-legal analysis to ascertain the cause and manner of death.

## 6. Conclusions

The presented review explored the role of miRNAs in the biomolecular mechanisms involved in TBI pathophysiology and their possible application in postmortem diagnosis in a forensic setting. Since discovery of their critical role in almost every biological function, miRNA profiling has attracted increasing interest in the clinical context to uncover the pathophysiological mechanisms of several diseases and provide suggestions for new therapeutic strategies. Translating miRNA profiling techniques to the forensic context has also taken on the utmost importance since they provide useful information, especially when implemented together with routine forensic investigations within a multidisciplinary approach.

Despite the great potential shown by these techniques, their active implementation in forensic investigations requires thorough validation studies to provide uniform and certain interpretation of the results and strong reliability for when the data are presented in a courtroom.

## Figures and Tables

**Table 1:** Main findings of the selected studies involving the role of miRNAs in TBI diagnosis (CSF = cerebrospinal fluid; mTBI = mild traumatic brain injury; mmTBI = mild to moderate traumatic brain injury; sTBI = severe traumatic brain injury; RT-PCR = real-time polymerase chain reaction).

Reference	Evaluated miRNAs	Detection Procedure	Matrix Used	Main Findings
Long et al. [24]	Exosome-derived miR-873a-5p	RT-PCR	Step 1: - In vitro stimulation of astrocytes with TBI extracts; - Isolation of astrocyte-derived exosomes, and exosome-derived miRNA profiling; - Addition of astrocyte-derived exosomes to cultured microglia. Step 2: - miRNA profiling on clinical brain samples from TBI patients (*n* = 15) who underwent neurosurgery, and comparison with in vitro study. Step 3: - In vitro study of miR-873a-5p-related pathway.	- miR-873a-5p is the most represented exosome-derived miRNA produced by TBI-stimulated astrocytes, and is involved in M2-polarization of microglia. A significant increase of miR-873a-5p is also observed in the necrotic areas of clinical human brain samples. miR-873a-5p guides the suppression of pro-inflammatory factors, and promotes the release of anti-inflammatory factors from the microglia.
Vorn et al. [25]	Up-regulated hsa-miR-520e, hsa-miR-499b-3p, hsa-miR-520b, hsa-miR-4488. Down-regulated hsa-miR-625-5p, hsa-miR-421, hsa-miR-664a-3p, hsa-miR-28-3p, hsa-miR-125a-5p, hsa-miR-222-3p, hsa-miR-140-5p, hsa-miR-98-5p, hsa-miR-148a-3p, hsa-miR-423-5p, hsa-miR-107, hsa-miR-181a-5p, hsa-miR-374a-5p, hsa-miR-340-5p, hsa-miR-29b-3p, hsa-miR-191-5p, hsa-miR-199a-3p, hsa-miR-126-3p, hsa-miR-23a-3p, hsa-miR-142-3p, hsa-miR-223-3p	Nano-sequencing	Plasma samples obtained from: - Patients with chronic mTBI (*n* = 29); - Healthy controls (*n* = 11).	- Differentially expressed exomiRNAs in chronic mTBI are involved in the mechanisms underlying TBI-related brain function impairment.
Rendell et al. [27]	miR-16	Microarray analysis and RT-PCR	Plasma samples obtained from: - sTBI patients (*n* = 10); - mTBI patients (*n* = 11); - Healthy controls (*n* = 10).	- 27 highly up-regulated miRNAs, out of which miR-16, miR-20a, miR-92a, miR-638, miR-765 were selected for known involvment in TBI pathophysiology and amplified by RT-PCR. All miRNAs appeared highly up-regulated in sTBI patients compared to healthy controls, with higher levels of miR-16 and miR-92a also compared to orthopedic injury controls.
	miR-20a			
	miR-92a			
	miR-638			
	miR-765			
Bhomia et al. [28]	miR-151-5p	RT-PCR	Serum samples obtained from: - sTBI patients (*n* = 8); - mmTBI patients (*n* = 8); - Orthopedic injury patients (*n* = 7); - Healthy controls (*n* = 8). CSF samples obtained from: - sTBI patients (*n* = 8); - Healthy controls (*n* = 6).	- miR-151-5p, miR-22 195, miR-20a, miR-30d, miR-328, miR-362-3p, miR-451, miR-486, miR-505*, and miR-92a appeared up-regulated in both mild/moderate and severe TBI patients compared to orthopedic injury patients and healthy controls. miR-195, miR-30d, miR-451, miR-92a, miR-486, miR-505, miR-362, miR-20a were significantly increased in TBI patients with abnormal CT scans (*n* = 12) compared to TBI patients with normal CT scans (*n* = 19). miR-328, miR-362-3p, miR-451, miR-486 were up-regulated in CSF samples of TBI patients.
	miR-22 195			
	miR-20a			
	miR-30d			
	miR-328			
	miR-362-3p			
	miR-451			
	miR-486			
	miR-505*			
	miR-92a			
Di Pietro et al. [29]	mTBI miR-425-5p, miR-126*, miR-144*, miR-590-3p, miR-624 sTBI miR-21, miR-335, miR-190, miR-193a-5p, miR-144*, and miR-625*	RT-PCR	Step 1: Serum samples collected 1 day and 15 days after injury from: - mTBI patients (*n* = 5); - sTBI patients (*n* = 5); - Healthy controls (*n* = 5). Step 2: Identification of miRNA changes that could differentiate between mild and severe TBI, using serum samples obtained from a wider causistry of 120 patients divided into 4 groups: - mTBI patients (*n* = 30); - sTBI patients (*n* = 30); - Extra-cranial injury (EC) control patients (*n* = 30); - Healthy controls (*n* = 30). All serum samples were collected at T_0_, T4–12 h, T48–72 h, and 15 days.	- Compared to healthy controls, in mTBI patients, changes were observed in the differential expression of 10 miRNAs at 1 day, and 15 miRNAs at 15 days; among these, only 5 miRNAs (miR-425-5p, miR-126*, miR-144*, miR-590-3p, miR-624) were similar at both time points. Compared to healthy controls, in sTBI patients a differential expression was observed for 19 miRNAs at 1 day, and 22 miRNAs at 15 days, with particular reference to miR-21, miR-335, miR-190, miR-193a-5p, miR-144*, and miR-625*.
Qin et al. [30]	miR-6867-5p	Microarray analysis and RT-PCR	Plasma samples obtained from: - Mild, moderate, and severe TBI patients (*n* = 90); - Healthy controls (*n* = 30).	- 7 miRNAs appeared commonly up-regulated while 6 miRNAs appeared commonly down-regulated between mild, moderate, and severe TBI. The RT-PCR evaluation ofmiR-6867-5p, miR-3665, miR-328-5p, miR-762, miR-3195, miR-4669, miR-2861 on a wider causistry revealed that, although all are up-regulated in all classes of TBI compared to controls: - miR-3195 and miR-328-5p levels are higher in sTBI compared to mild and moderate TBI; - miR-6867 levels are higher in moderate and severe TBI compared to mild TBI.
	miR-3665			
	miR-328-5p			
	miR-762			
	miR-3195			
	miR-4669			
	miR-2861			
Ma et al. [31]	miR-18a	RT-PCR	Serum samples obtained from sTBI patients (*n* = 20) at 2–12–24–48–72 h from trauma.	- miR-18a, miR-203, miR-146a, miR-149, miR-23b, and miR-let-7b → >10-fold increase at 12 h compared to 2 h time-point; - miR-203, miR-146a, miR-149, miR-23b, and miR-let-7b → >10-fold increase also at 24 h; - miR-181d, miR-29a, and miR-18b → >5-fold increase at 48 h; - miR-203, miR-146a, and miR-149 → >5-fold increase after 72 h.
	miR-203			
	miR-146a			
	miR-149			
	miR-23b			
	miR-let-7b			
	miR-181d			
	miR-29a			
	miR-18b			
Yan et al. [32]	miR-219a-5p	Microarray analysis and RT-PCR	Step 1: miRNA microarray on serum samples obtained from: - sTBI patients (*n* = 15); - Healthy controls (*n* = 15). Step 2: RT-PCR on serum samples obtained from: - sTBI patients (*n* = 81); - Moderate TBI patients (*n* = 81); - Healthy controls (*n* = 82). Step 3: - TBI patients without head CT lesions (*n* = 26); - TBI patients with head CT lesions (*n* = 136).	- miR-103a-3p, miR-219a-5p, miR-302d-3p, miR-422a, miR-518f-3p, miR-520d-3p, and miR-627 were significantly up-regulated in both sTBI and mTBI patients compared to controls; among these, miR-219a-5p, miR-422a, and miR-520d-3p levels were significantly higher in sTBI patients compared to mTBI patients.
	miR-422a			
	miR520d-3p			
	miR-103a-3p			
	miR302d-3p			
	miR-518f-3p			
	miR-627			
Schindler et al. [33]	miR-9-5p	RT-PCR	Blood samples obtained from: - Injured patients with TBI (PT + TBI, *n* = 13); - Injured patients without TBI (PT, *n* = 8); - Patients with isolated TBI (isTBI, *n* = 12).	- miR-9-5p, miR-142-3p, and miR-219a-5p were not detected in any group; - miR-338-3p levels were the same in each trauma group; - miR-423-3p significantly increased in isTBI patients, immediately followed by PT + TBI patients, compared to PT group; - Statistical analyses further showed that miR-423-3p levels positively correlate with TBI severity and risk of mortality.
	miR-124-3p			
	miR-162-3p			
	miR-219a-5p			
	miR-338-3p			
	miR-423-3p			

**Table 2:** Summary of the main advantages and limits of miRNA profiling application to the post mortem diagnosis of TBI.

Advantages	Limits
- Detectable in highly degraded samples; - High stability at extreme temperatures, pH conditions and chemical treatments; - Preserved from endogenous RNAse activity; - Easily evaluable in fluid samples; - Highly sensitive detection procedures (microarray, NGS, qRT-PCR).	- Lack of uniform and validated detection protocols; - Lack of standard endogenous controls and normalization procedures; - Variability related to demographic features (age, sex, BMI); - Lack of procedures aimed at avoiding contamination or miRNA profiling alterations; - Cost/effectivness issues in daily forensic practice.

## Data Availability

No new data were created.

## References

[1] Ros A.C., Bacci S., Luna A., Legaz I. (2022). Forensic Impact of the Omics Science Involved in the Wound: A Systematic Review. Front. Med..

[2] Glynn G.L. (2020). Potential Applications of microRNA Profiling to Forensic Investigations. RNA.

[3] Rocchi A., Chiti E., Maiese A., Turillazzi E., Spinetti I. (2020). MicroRNAs: An Update of Applications in Forensic Science. Diagnostics.

[4] Bartel D.P. (2009). MicroRNAs: Target Recognition and Regulatory Functions. Cell.

[5] Hanson E.K., Lubenow H., Ballantyne J. (2009). Identification of forensically relevant body fluids using a panel of differentially expressed microRNAs. Anal. Biochem..

[6] Sessa F., Maglietta F., Bertozzi G., Salerno M., Di Mizio G., Messina G., Montana A., Ricci P., Pomara C. (2019). Human Brain Injury and miRNAs: An Experimental Study. Int. J. Mol. Sci..

[7] Pennisi G., Torrisi M., Cocimano G., Esposito M., Salerno M., Sessa F. (2022). Vitality markers in forensic investigations: A literature review. Forensic Sci. Med. Pathol..

[8] Najem D., Rennie K., Ribecco-Lutkiewicz M., Ly D., Haukenfrers J., Liu Q., Nzau M., Fraser D.D., Bani-Yaghoub M. (2018). Traumatic brain injury: Classification, models, and markers. Biochem. Cell Biol..

[9] Balakathiresan N., Bhomia M., Chandran R., Chavko M., McCarron R.M., Maheshwari R.K. (2012). MicroRNA Let-7i Is a Promising Serum Biomarker for Blast-Induced Traumatic Brain Injury. J. Neurotrauma.

[10] Chandran R., Sharma A., Bhomia M., Balakathiresan N.S., Knollmann-Ritschel B.E., Maheshwari R.K. (2017). Differential expression of microRNAs in the brains of mice subjected to increasing grade of mild traumatic brain injury. Brain Inj..

[11] Johnson D., Cartagena C.M., Tortella F.C., Dave J.R., Schmid K.E., Boutté A.M. (2017). Acute and subacute microRNA dysregulation is associated with cytokine responses in the rodent model of penetrating ballistic-like brain injury. J. Trauma Acute Care Surg..

[12] Zhao J., Qu Y., Wu J., Cao M., Ferriero D.M., Zhang L., Mu D. (2013). PTEN inhibition prevents rat cortical neuron injury after hypoxia–ischemia. Neuroscience.

[13] Han Z., Chen F., Ge X., Tan J., Lei P., Zhang J. (2014). miR-21 alleviated apoptosis of cortical neurons through promoting PTEN-Akt signaling pathway in vitro after experimental traumatic brain injury. Brain Res..

[14] Ge X., Han Z., Chen F., Wang H., Zhang B., Jiang R., Lei P., Zhang J. (2015). miR-21 alleviates secondary blood–brain barrier damage after traumatic brain injury in rats. Brain Res..

[15] Ohtake Y., Hayat U., Li S. (2015). PTEN inhibition and axon regeneration and neural repair. Neural Regen. Res..

[16] Zwirner J., Kulakofsky R., Fitzek A., Schröder A.S., Bohnert S., Franke H., Renné T., Tse R., Ondruschka B. (2022). Forensic biomarkers of lethal traumatic brain injury. Int. J. Leg. Med..

[17] Ghaith H.S., Nawar A.A., Gabra M.D., Abdelrahman M.E., Nafady M.H., Bahbah E.I., Ebada M.A., Ashraf G.M., Negida A., Barreto G.E. (2022). A Literature Review of Traumatic Brain Injury Biomarkers. Mol. Neurobiol..

[18] Wang J., Wang J., Li X., Shu K. (2022). Cell-Derived Exosomes as Therapeutic Strategies and Exosome-Derived microRNAs as Biomarkers for Traumatic Brain Injury. J. Clin. Med..

[19] Cente M., Matyasova K., Csicsatkova N., Tomikova A., Porubska S., Niu Y., Majdan M., Filipcik P., Jurisica I. (2022). Traumatic MicroRNAs: Deconvolving the Signal After Severe Traumatic Brain Injury. Cell. Mol. Neurobiol..

[20] Bertozzi G., Maglietta F., Sessa F., Scoto E., Cipolloni L., Di Mizio G., Salerno M., Pomara C. (2020). Traumatic Brain Injury: A ForensicApproach: A Literature Review. Curr. Neuropharmacol..

[21] Panaro M.A., Benameur T., Porro C. (2020). Extracellular Vesicles miRNA Cargo for Microglia Polarization in Traumatic Brain Injury. Biomolecules.

[22] Guedes V.A., Devoto C., Leete J., Sass D., Acott J.D., Mithani S., Gill J.M. (2020). Extracellular Vesicle Proteins and MicroRNAs as Biomarkers for Traumatic Brain Injury. Front. Neurol..

[23] Vaughn M.N., Winston C.N., Levin N., Rissman R.A., Risbrough V.B. (2022). Developing Biomarkers of Mild Traumatic Brain Injury: Promise and Progress of CNS-Derived Exosomes. Front. Neurol..

[24] Long X., Yao X., Jiang Q., Yang Y., He X., Tian W., Zhao K., Zhang H. (2020). Astrocyte-derived exosomes enriched with miR-873a-5p inhibit neuroinflammation via microglia phenotype modulation after traumatic brain injury. J. Neuroinflamm..

[25] Vorn R., Suarez M., White J.C., Martin C.A., Kim H.-S., Lai C., Yun S.-J., Gill J.M., Lee H. (2021). Exosomal microRNA Differential Expression in Plasma of Young Adults with Chronic Mild Traumatic Brain Injury and Healthy Control. Biomedicines.

[26] Toffolo K., Osei J., Kelly W., Poulsen A., Donahue K., Wang J., Hunter M., Bard J., Wang J., Poulsen D. (2019). Circulating microRNAs as biomarkers in traumatic brain injury. Neuropharmacology.

[27] Redell J.B., Moore A.N., Ward N.H., Hergenroeder G.W., Dash P.K. (2010). Human Traumatic Brain Injury Alters Plasma microRNA Levels. J. Neurotrauma.

[28] Bhomia M., Balakathiresan N.S., Wang K.K., Papa L., Maheshwari R.K. (2016). A Panel of Serum MiRNA Biomarkers for the Diagnosis of Severe to Mild Traumatic Brain Injury in Humans. Sci. Rep..

[29] Di Pietro V., Ragusa M., Davies D., Su Z., Hazeldine J., Lazzarino G., Hill L.J., Crombie N., Foster M., Purrello M. (2017). MicroRNAs as Novel Biomarkers for the Diagnosis and Prognosis of Mild and Severe Traumatic Brain Injury. J. Neurotrauma.

[30] Qin X., Li L., Lv Q., Shu Q., Zhang Y., Wang Y. (2018). Expression profile of plasma microRNAs and their roles in diagnosis of mild to severe traumatic brain injury. PLoS ONE.

[31] Ma S.-Q., Xu X.-X., He Z.-Z., Li X.-H., Luo J.-M. (2019). Dynamic changes in peripheral blood-targeted miRNA expression profiles in patients with severe traumatic brain injury at high altitude. Mil. Med Res..

[32] Yan J., Bu X., Li Z., Wu J., Wang C., Li D., Song J., Wang J. (2019). Screening the expression of several miRNAs from TaqMan Low Density Array in traumatic brain injury: miR-219a-5p regulates neuronal apoptosis by modulating CCNA2 and CACUL1. J. Neurochem..

[33] Schindler C.R., Woschek M., Vollrath J.T., Kontradowitz K., Lustenberger T., Störmann P., Marzi I., Henrich D. (2020). miR-142-3p Expression Is Predictive for Severe Traumatic Brain Injury (TBI) in Trauma Patients. Int. J. Mol. Sci..

[34] Lo Re G., Argo A., Midiri M., Cattaneo C. (2020). Radiology in Forensic Medicine: From Identification to Post-Mortem Imaging.

[35] Argo A., Zerbo S., Buscemi R., Trignano C., Bertol E., Albano G.D., Vaiano F. (2022). A Forensic Diagnostic Algorithm for Drug-Related Deaths: A Case Series. Toxics.

[36] Iarussi F., Cipolloni L., Bertozzi G., Sasso L., Ferrara M., Salerno M., Rubino G.T.R., Maglietta F., Dinisi A., Albano D. (2020). Dog-bite-related attacks: A new forensic approach. Forensic Sci. Int..

[37] O’Connell G.C., Smothers C.G., Winkelman C. (2020). Bioinformatic analysis of brain-specific miRNAs for identification of candidate traumatic brain injury blood biomarkers. Brain Inj..

